# Malaria knowledge, attitude, and practice among communities involved in a seasonal malaria chemoprevention study in Nanyumbu and Masasi districts, Tanzania

**DOI:** 10.3389/fpubh.2023.976354

**Published:** 2023-02-15

**Authors:** Billy Ngasala, Richard O. Mwaiswelo, Frank Chacky, Fabrizio Molteni, Ally Mohamed, Samwel Lazaro, Bushukatale Samuel, Bruno P. Mmbando

**Affiliations:** ^1^Department of Parasitology and Medical Entomology, Muhimbili University of Health and Allied Sciences, Dar es Salaam, Tanzania; ^2^Department of Microbiology, Immunology and Parasitology, Hubert Kairuki Memorial University, Dar es Salaam, Tanzania; ^3^National Malaria Control Programme, Ministry of Health, Community Development, Gender, Elderly and Children, Dodoma, Tanzania; ^4^Tanga Research Centre, National Institute for Medical Research, Tanga, Tanzania

**Keywords:** knowledge, attitude, utilization, malaria control, Masasi, Nanyumbu, Tanzania

## Abstract

**Background:**

Utilization of malaria interventions is influenced by, among other things, the level of knowledge and attitude that the community has toward the infection as well as the available interventions. This study assessed malaria knowledge, attitudes, and practices on malaria infection and interventions in Masasi and Nanyumbu districts, Tanzania.

**Methods:**

A community-based cross-sectional survey was conducted between August and September 2020, among the heads of households having at least one under-five child. Information on knowledge, attitudes, and practices on malaria infection and interventions was gathered from the heads of the households using a structured questionnaire. The knowledge level was classified into low, moderate, and high. Attitudes were classified into positive and negative, whereas the practices were classified into good and poor. Children aged between 3 and 59 months were screened for malaria infection using a malaria rapid diagnostic test (mRDT). The proportion of the households' heads with high level of knowledge was the primary outcome. Proportions were compared using *Chi*-square or fisher's test, and logistic regression analysis was used as appropriate.

**Results:**

A total of 1,556 household heads were interviewed, 1,167 (75.00%) were male, and according to marital status, 1,067 (68.57%) were couples. All the household heads had some knowledge of malaria, but 47.33% (736/1,555) and 13.83% (215/1,555) of them had moderate and high knowledge, respectively. The level of knowledge on malaria was significantly influenced by gender [adjusted odds ratio (aOR) = 0.72, 95.00% confidence interval (CI) = 0.56–0.94, *p* = 0.017], level of education (aOR = 1.50, 95.00% CI = 1.04–2.16, *p* = 0.03), and the occupation of the household head (aOR = 1.90, 95.00% CI = 1.22–2.96, *p* = 0.004). Majority of the households [83.87% (1,305/1,556)] had bed nets hanging on the sleeping spaces. Of the household heads possessing bed nets, 85.10% (514/604), 79.62% (586/736), and 95.35% (205/215) of them had a low, moderate, and high level of knowledge on malaria infection, respectively (trend *x*^2^ = 31.53, *p* < 0.001). The majority [95.04% (1,474/1,551)] of the household heads perceived sleeping under the bed net to be beneficial. Furthermore, 15.56% (94/604), 14.67% (108/736), and 7.44% (16/215) of the household heads with low, moderate, and high knowledge, respectively, had children with malaria infection (trend *x*^2^ = 9.172, *p* = 0.01).

**Conclusion:**

The study population had a good level of knowledge about malaria infection, and a good attitude toward malaria interventions, and the majority of them were using bed nets.

## Background

Despite the achievements performed by Tanzania in recent years, the malaria infection remains as one of the leading causes of morbidity and mortality in the country (1, 2). Malaria prevalence in the country has declined to 7.31%, however, it varies from one region or district to another, with some regions having prevalence as low as one percent while others show a higher prevalence up to 40.00% (1). On a global scale, Tanzania is still among 10 countries with the highest prevalence of malaria (3–5), and in 2020 this country contributed to about 5.00% of the global malaria-related deaths (3).

Tanzania recently included the seasonal malaria chemoprevention (SMC) strategy in the 2020–2025 Malaria Control Strategic Plan (6, 7). However, before it was scaled out, the protective effectiveness of the strategy had to be evaluated in Nanyumbu and Masasi districts, the settings where malaria transmission showed to be highly seasonal. It was also important that the target communities' level of knowledge, attitudes, and practices on malaria infection and the available interventions was assessed. The information would help to understand how SMC would be perceived and utilized in the two settings. Likewise, understanding the communities' level of knowledge, attitudes, and practices would enable them to improve the implementation of the available interventions and hence to increase their impact against the infection.

Major malaria interventions in Tanzania include vector control using insecticide-treated bed nets (ITNs) and indoor residual spraying (IRS), clinical case management using artemisinin-based combination therapy (ACT), and intermittent preventive treatments in pregnancy (IPTp) using sulphadoxine-pyrimethamine (SP) (1, 6, 7). The uptake of these control measures at the community level is, however, influenced by factors such as the community level of knowledge on malaria infection and its interventions. Studies have shown high knowledge of malaria infection and its interventions, and a positive attitude toward the interventions to be associated with higher coverage and utilization of the interventions in the community and hence higher impact of the interventions (8–12). Nonetheless, although knowledge improves awareness, it does not always transform into positive attitudes and practices. This has been demonstrated in some studies whereby high knowledge of malaria infection and its interventions, and the ownership of control tools were not correlating with the utilization of the tools (13–16). In addition, knowledge, attitudes, and practices are influenced by several socioeconomic factors. Studies have shown that knowledge of malaria infection, attitude toward, and utilization of malaria interventions to be influenced by the level of education one has attained (10, 14, 17–22), availability of health facilities (9), marital status (19, 21), gravidity (9, 21), age (20, 23), employment status (20), and urban settlement (19, 24). Utilization of the interventions, such as ITNs, is also influenced by factors including its availability (15), household size (23), ITNs discomforts for instance irritation and bed net smell (15), availability of traditional methods such as herbs that wade off mosquitoes, type of housing, weather conditions (9), and socioeconomic status (15, 23). The ITNs, management of clinical malaria cases using ACT, and IPTp-SP are the major malaria interventions in Masasi and Nanyumbu districts. However, the communities' level of knowledge on malaria infection and interventions, attitudes toward malaria interventions, and level of utilization of the available interventions, its influencing factors, and how in turn influences malaria infection in Masasi and Nanyumbu districts are not well-understood. This study, therefore, aimed to assess the level of knowledge, attitudes, and practices (KAP) on malaria infection and utilization of the available interventions among communities in Masasi and Nanyumbu districts, Tanzania, before the implementation of SMC. The socioeconomic factors influencing KAP, and the prevalence of malaria among under-five children were also determined.

## Materials and methods

### Study area

**Figure 1:**
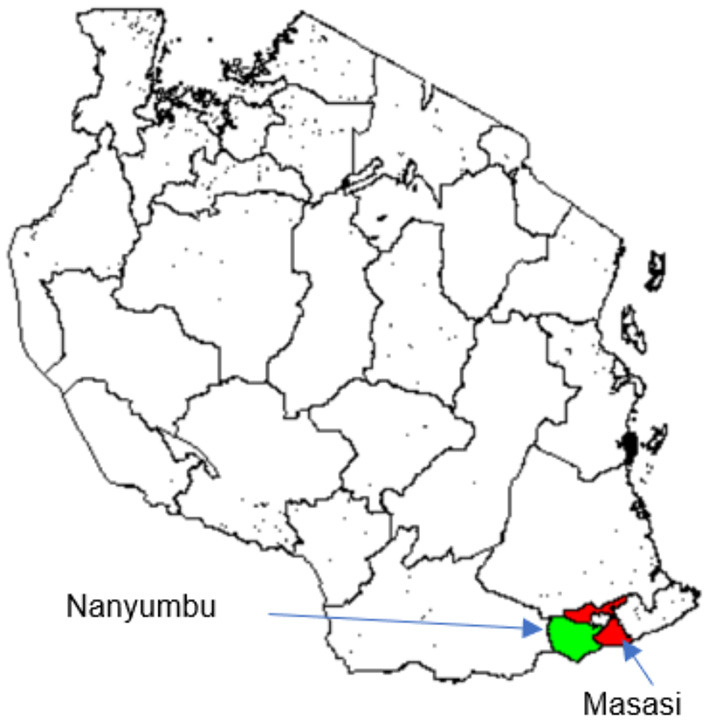
Map of Tanzania showing location of the study districts, Masasi (red) and Nanyumbu (green).

The study was carried out in Nanyumbu and Masasi districts, Mtwara region between August and September, 2020 (Figure 1). Nanyumbu is divided into 14 wards and 89 villages, whereas Masasi has 22 wards and 159 villages. Nanyumbu district has a total population of 166,277, and Masasi has 269,590. More than half of the population in both districts lives in rural areas. In 2018, there were 44,319 and 73,998 households in Nanyumbu and Masasi districts, respectively. Major economic activities in Masasi and Nanyumbu districts include subsistence farming, petty trade, fishing, and small-scale mining.

Nanyumbu and Masasi districts have annual rainfall averaging 939 mm and a temperature ranging between 15.60°C and 32.20°C. The rainy season in Nanyumbu and Masasi districts is between January and April. Both districts have highly seasonal malaria transmissions where more than 60.00% of cases occur between March and July. *Plasmodium falciparum* is a predominant malaria parasite, and *Anopheles arabiensis* a major vector. Insecticide-treated bed-nets, and diagnosis of the malaria infection using malaria rapid diagnostic test (mRDT) and treatment of clinical cases using artemisinin-based combination therapy are the major malaria control measures (6).

### Study design

This was a baseline cross-sectional survey, part of a community-based open cluster-randomized study involving two arms (interventional and control) using wards as a randomization unit. The clusters were randomized using Research Randomizer version 4 computer software (Wesleyan University, Connecticut, USA) (25). A ward instead of a district was chosen as a sampling unit to reduce heterogeneity in rates of malaria infections that could be observed between the two districts, which would have been associated with factors that might have not been accounted for during the study. In each of the study wards, a health facility nearest to the centroid of the ward was selected as an evaluation point for study participants within the catchment area. A study village(s) within the health facility catchment area was identified (satellite villages). In each of the catchment villages, a village leader was requested to prepare a register of households with at least one under-five children. The registers were used to visit all the households with under-five children to collect the demographic as well as socio-economic characteristics of the households. Screening for malaria morbidity among the under-five children was carried out at the satellite health facilities whereby caregivers were requested to bring all the febrile and afebrile children of the required age for clinical and laboratory assessments.

### Study population

The heads of the households having at least one under-five children were involved in the household surveys to assess knowledge, attitudes, practices, and socioeconomic status, whereas children were screened for malaria infection. Inclusion criteria were: being head of the household, living within the catchment area for the past 5 years, having a child aged between 3 and 59 months, and willingness to participate in the study. The exclusion criteria were: children with severe illness, a history of intake of antimalarial drugs within the past 30 days, or a child being under cotrimoxazole prophylaxis.

The head of the household and the household itself were defined as previously (26). In the absence of the head of the household, a responsible person above 18 years old who had been appointed by the family was interviewed.

### Procedures for data collection

Research assistants including clinicians, laboratory technicians, and community health workers (CHWs) were trained on the study objectives and procedures including obtaining the informed consent, administering the questionnaire, measuring the axillary temperature, height, and weight, taking the peripheral finger-prick blood samples, preparation of thick and thin smears, and use of mRDTs, before data collection. Mobilization of the participating communities was organized through local meetings and announcements in the houses of worship before the start of the study.

#### Assessment of knowledge, attitude, practice, and socioeconomic status

A structured questionnaire with both close and open-ended questions, developed in English and then translated into Kiswahili was used to assess the knowledge, attitudes, and practices of the heads of households toward malaria infection and interventions, and the household socioeconomic status. The CHWs administered the questionnaire which inquired information on demographic characteristics of the household, socioeconomic status including house type and household assets, knowledge on malaria infection (e.g., its transmission, and symptoms) and its interventions, as well as ownership, usage, and attitude of the household on the available major malaria interventions in the districts, particularly insecticide-treated bed nets (ITNs). The use of ITNs was assessed by asking if the household occupants slept under ITNs the previous night, and the CHWs confirmed the presence of the bed nets.

#### Malaria screening

A peripheral finger-prick blood sample was used to determine the presence of malaria infection using the malaria rapid diagnostic test (mRDT), and thick and thin films for microscopy as previously described (26). Briefly, the mRDTs were read within 15 min. Thin films were fixed using methanol. Both thin and thick blood films were air-dried, stained using 3.0% Giemsa stain for 1 h, and examined for malaria parasites at 100 × high power fields under immersion oil before a film was declared negative.

### Study outcomes

The primary outcome was the prevalence of heads of households with high knowledge of malaria infection and its interventions. Secondary outcomes included the (i) prevalence of heads of the household following good practices for malaria interventions, (ii) prevalence of heads of households having good attitudes toward malaria interventions, (iii) prevalence of heads of households having knowledge on malaria interventions and practices, (iv) socioeconomic factors influencing knowledge and practices on malaria intervention, and (v) prevalence of malaria infection among children from households with knowledgeable heads on malaria infection and its interventions.

### Ethical consideration

Approval to conduct the study was obtained from the Ethics Committee of the Muhimbili University of Health and Allied Sciences. During the conduct of the study, the declaration of Helsinki, good clinical practices, and regulations in Tanzania were followed.

Meetings were held with community, administrative, and religious leaders to explain the study motives and activities, and also sought community approval. The CHWs also visited the households to explain the study motives, provided information sheets, and sought signed consent from the heads of households.

### Statistical analysis

This was a baseline survey for seasonal malaria chemoprevention (SMC) study, thus sample size calculation was based on the estimations made for SMC and are presented elsewhere (26). Briefly, a total of 20 wards were selected for the study, and in each ward 106 children were to be assessed with a power of 80.00% and an alpha (type one error) of 0.05. An attrition rate of 20.00% was considered resulting in a sample of 128 children per evaluable ward. Similar to the number of children expected to participate in the screening of malaria infection, 128 households had to be assessed for heads of household knowledge, attitude, practice, and socioeconomic information assuming each household contributed one child.

Data were collected electronically in open data kit software using tablet computers. Quality control and assurance of the data were maintained at all stages of data collection to archiving. Back-ups of data were made daily onto external hard disks and stored in a secured place separately from the building hosting data management section. Source documents (in case of paper-based) were achieved and sprayed regularly to keep them away from destruction by insects. Data were cleaned and analyzed using the Statistical Package for Social Sciences (SPSS) version 20. The knowledge, attitudes, and practices scores of the participants were determined as previously described elsewhere (27). Briefly, for knowledge assessment, a correct response to each question was given a score of 1, and a wrong or unsure response was given a score of 0. The original Bloom's cut-off points score was used to categorize the level of knowledge into low, moderate, and high based on the correct responses as follows: a score of ≤ 59.00% meant low knowledge; a score of 60.00–79.00% designated as moderate knowledge, and a score of 80.00–100.00% indicated high knowledge. Furthermore, the level of knowledge was also grouped into two categories (low vs. moderate to high) where the latter grouped the moderate-high knowledge scores. On the other hand, the attitude was assessed using Likert's scaling technique. The questions on Likert's scaling had positive and negative responses that ranged from strongly agree (score 5), agree (score 4), undecided (score 3), disagree (score 2), to strongly disagree (score 1). The responses were summed up, and a total score was obtained for each respondent. The mean score was calculated, and respondents with a score of greater than or equal to the mean score (4.14) were considered as having a positive attitude whereas those with a score of less than the mean score (4.14) were taken as having a negative attitude. Conversely, practices were also assessed using Likert's scaling method. The scoring of responses ranged from never (score 0), sometimes (score 1), to always (score 2). The responses were summed up, a total score was obtained for each respondent, and the mean practice score was computed across all the study participants. An individual was assumed to have good practice when his/her overall practice score was equal to/ or more than the mean practice score (1.03), whereas, was considered as having poor practice when his/her overall practice score was less than the mean practice score (1.03).

Categorical variables were presented in proportions and compared using *Chi*-square/fishers' tests. Logistic regression analysis was used to assess the association of socioeconomic factors with knowledge of malaria infection and interventions. A *p*-value < 0.05 was considered statistically significant.

## Results

### Baseline characteristics of the study population

A total of 1,556 household heads were interviewed. The baseline characteristics of the study participants are presented in Table 1. The majority of the heads of the households were male [75.00% (1,167/1,556)], had primary education [82.71% (1,287/1,556)], and were farmers [92.99% (1,447/1,556)]. Also, the majority of the households had five or more occupants [79.24% (1,233/1,556)].

**Table 1:** Baseline characteristics of the households.

**Variable**	**Statistics**
Age, median (interquartile range), years	34 (28–42)
Sex, male, *n* (%)	1,167 (75.00)
**Marital status**, ***n*** **(%)**	
Couple	1,067 (68.60)
Single	410 (26.40)
Divorced	78 (5.01)
**Level of education**, ***n*** **(%)**	
None	131 (8.40)
Primary	1,287 (82.70)
Secondary	138 (8.90)
**Occupation**, ***n*** **(%)**	
Farmer	1,447 (93.80)
Petty trader	96 (6.20)
**Number of the household occupant**, ***n*** **(%)**	
≤ 4	318 (20.50)
≥5	1,233 (79.50)
**Number of under-fives in a household**, ***n*** **(%)**	
1	1,232 (82.30)
≥2	265 (17.70)
**Socioeconomic status**, ***n*** **(%)**	
Low (0–33.3th)	369 (32.20)
Middle (33.4–66.7th)	382 (33.30)
Upper (67.8–100th)	395 (34.50)

### Study population knowledge of malaria

The knowledge of the study participants on malaria transmission, symptoms, and prevention is presented in Table 2. Overall, 38.82% (604/1,555), 47.33% (736/1,555), and 13.83% (215/1,555) of the household heads showed low, moderate, and high knowledge of malaria infection, respectively.

**Table 2:** Study population knowledge of malaria infection.

**Variable**	**Frequency (%)**
**Malaria transmission**	
Mosquito bite	1,434 (92.20)
Don't know	28 (1.80)
**Mosquitos breeding sites**	
Stagnant water	1,297 (83.40)
Grasses	570 (36.60)
Bushes	534 (34.30)
Don't know	71 (4.60)
**Malaria symptoms**	
Fever	1,152 (74.00)
Headache	1,008 (64.80)
Sweating	296 (19.01)
Shivering	560 (36.03)
Body weakness	606 (38.90)
Abdominal pain	287 (18.40)
**Malaria control measures**	
Treated bed net	1,472 (94.60)
Insecticide spray	455 (29.20)
Destroying breeding sites	165 (10.60)
Mosquito coil repellents	203 (13.01)
Treatment of the malaria patients	357 (22.90)
Don't know	7 (0.40)

### Socioeconomic factors influencing knowledge on malaria infection

The relationship between socioeconomic factors and knowledge as a binary variable (low knowledge vs. moderate to high) is presented in Table 3. In univariate analysis sex of the household head, the level of education of the household head especially the primary school level and the occupation of the household head were the factors significantly associated with having a high level of knowledge of the infection. Likewise, in the multivariate analysis, the same factors were significantly associated with knowledge of malaria infection.

**Table 3:** Association of socioeconomic factors with knowledge of malaria infection.

**Variable**	**Frequency (%)**	**Univariate**	**Multivariate**
		**OR (95%CI);** ***p*****-value**	**aOR (95%CI)** ***p*****-value**
District—Masasi	1,057 (67.90)	1	
Nanyumbu	499 (32.10)	1.15 (0.93–1.44), 0.20	
**Age of head of household**			
<45 years	1,254 (80.60)	1	
≥45 years	302 (19.40)	1.04 (0.80–1.35), 0.76	
**Sex of head of household**			
Male	1,167 (75.00)	1	1
Female	389 (25.00)	0.75 (0.60–0.95), 0.020	0.72 (0.56–0.94), 0.02
**Marital status**			
Single	410 (26.30)	1	
Couple	1,067 (68.60)	0.89 (0.70–1.12), 0.31	0.76 (0.59–0.99), 0.04
Others (divorced/widow etc.)	79 (5.10)	0.65 (0.40–1.05), 0.08	0.74 (0.45–1.22), 0.24
**Level of education**			
None	131 (8.40)	1	1
Primary education	1,287 (82.70)	1.50 (1.04–2.15), 0.03	1.50 (1.04–2.16), 0.03
Secondary education or higher	138 (8.90)	1.38 (0.85–2.23), 0.19	1.07 (0.64–1.80), 0.79
**Occupation**			
Subsistence farmer	1,428 (91.80)	1	
Others	128 (8.20)	1.76 (1.18–2.64), 0.01	1.90 (1.22–2.96), 0.004
**Household size**			
<5	892 (57.50)	1	
≥5	659 (42.50)	1.06 (0.86–1.30) 0.60	
**Number of under-fives**			
1	1,232 (79.20)	1	
≥2	323 (20.80)	1.06 (0.82–1.36), 0.65	

OR, odds ratio; aOR, adjusted odds ratio; CI, confidence interval.

### Malaria control and prevention practices in the study population

Bed nets [95.89% (1,492/1,556)], insecticide spray [24.68% (384/1,556)], and mosquito coil [16.35% (255/1,556)] were the major tools mentioned by the population to be used for the prevention of the infection. But when the sleeping spaces were inspected, a slightly less percentage [83.87% (1,305/1,556)] of the households had bed nets hanging on the spaces. The major sources of the bed nets were antenatal clinics [78.39% (1,023/1,305)], bed nets free distribution mass campaign [38.10% (497/1,305)], school programs [14.48% (189/1,305)], and the retail shop [8.89% (116/1,305)]. Of the bed nets, 66.51% (971/1,460) were long-lasting treated nets (LLINs), 1.16% (17/1,460) regularly treated bed nets, 7.95% (116/1,460) untreated nets, and 6.10% (89/1,460) were not known. Conversely, the high cost of bed nets [62.98% (131/208)], scarcity of bed nets [60.10% (125/208)], reduced air circulation [5.29% (11/208)], increased heat [3.36% (7/208)], and lack of protection 0.48% (1/208) were the reasons for some households failing to possess and/ or using bed nets.

### Relationship between knowledge, bed net ownership, and use, and malaria infection

Eighty-four per cent (1,305/1,556) of the households had bed nets. Of the households with bed nets, 85.10% (514/604), 79.61% (586/736), and 95.35% (205/215) of their heads had a low, moderate, and high level of knowledge of malaria infection, respectively. The high level of knowledge on the infection was significantly associated with the ownership of bed nets (trend *x*^2^ = 31.53, *p* < 0.001). Moreover, 96.86% (1,264/1,305) of the household heads reported having slept under the bed net the previous night. But there was no statistically significant difference in the proportions of household heads with low [96.11% (494/514)], moderate [96.93% (568/586)], or high [98.50% (202/205)] knowledge of the infection who slept under the bed net in the previous night (trend *x*^2^ = 2.86, *p* = 0.24). On the other hand, 34 household heads reported to have not slept under the bed net the previous night. Not having a bed net [58.82% (20/34)], negligence [17.64% (6/34)], bed net increasing temperature [11.76% (4/34)], dirty bed nets [5.88% (2/34)], absence of mosquitoes [2.94% (1/34)], and attending the funeral [2.94% (1/34)] were the reasons for not using the bed net in the previous night.

Out of 2,340 screened children, 373 (15.94%) had malaria infection by mRDT. Furthermore, 15.56% (94/604), 14.67% (108/736), and 7.44% (16/215) of the household heads with low, medium, and higher knowledge, respectively, had children with malaria infection. High knowledge of malaria infection was significantly associated with a low prevalence of the infection (trend *x*^2^ = 9.17, *p* = 0.01).

### Study population attitude toward bed net use

Ninety-five per cent (1,474/1,551) of the household heads perceived sleeping under the bed net to be beneficial, and also 98.81% (1,080/1,093) of them felt comfortable sleeping under the bed net. Protection against mosquito bite [90.20% (1,177/1,305)], protection against malaria [60.50% (790/1,305)], and protection against insects [23.60% (308/1,305)] were mentioned as benefits of using the bed nets, whereas 2.76% (36/1,305) of the household heads thought the use of bed net as just a habit. Furthermore, 99.18% (1,084/1,093) of the household heads were ready to get new bed nets from any source in case the ones they had would become unusable. Likewise, 98.81% (1,080/1,093) of the household heads were ready to advise others to use bed nets.

## Discussion

This study aimed to assess the knowledge, attitudes, and practices on malaria infection and its interventions in the communities of Masasi and Nanyumbu districts, Tanzania, involved in the seasonal malaria chemoprevention. The findings showed that the majority of the study population presented good knowledge of malaria infection and interventions. Similar findings have been reported in other studies (14, 28–31). Of the study participants, 92.20% knew that malaria is transmitted through a mosquito bite. Findings in other parts of Tanzania (17) and other countries (32) have indicated a much lower proportion of subjects having knowledge of malaria transmission. Likewise, 83.40% of the study population knew that mosquitoes breed in stagnant water, but only 36.60 and 34.30% knew that mosquitoes could thrive in grasses and bushes, respectively. Fever was the most mentioned malaria symptom, followed by headache; however, other symptoms including sweating, shivering, body weakness, and abdominal pain were each mentioned by <40.00% of the individuals. Furthermore, treated bed nets were mentioned by 94.60% of the study population as a malaria intervention, but majority of them did not know other interventions such as insecticide spray, destroying of breeding sites, mosquito coil repellents, as well as treatment of malaria patients. The smooth implementation and uptake of malaria interventions in the community and individual households are dependent on among other factors the knowledge of malaria infection and its control measures an individual has and in turn influences attitude toward and the utilization of the available interventions. It is clear that the population in this study was knowledgeable on some components of malaria infection and interventions, and this could explain why the overall level of knowledge was moderate. There is a need to improve knowledge on malaria infection and intervention in this study population.

On the other hand, the gender of the household head (female), level of education, and occupations were the factors significantly positively associated with high knowledge. Findings in other studies have also indicated that the level of education (10, 14, 17–22), and some occupations (20) are associated with knowledge of malaria infection. Nonetheless, while other studies have shown that marital status (19, 21), and age (20, 23) of the household head are associated with high knowledge, in this study these factors were not associated with high knowledge of malaria infection. Of note, in this study population, the household heads' low level of knowledge of malaria infection and intervention was significantly associated with having children with malaria infection. Probably, due to their low knowledge of the importance of bed nets and other interventions, the household heads failed to see the importance of having bed nets and other interventions or replacing them once they are dilapidated, thus predisposing their children to malaria infection. This observation additionally emphasizes the need to continue with educational interventions when distributing bed nets to the communities.

The majority of the household heads (95.90%) mentioned bed nets as one of the major malaria interventions in their households, but on inspection only 84.00% of the households had bed nets hung on the sleeping spaces. A slightly lower proportion of the population owning bed nets has been reported in other parts of Tanzania (17) and other countries (29–31). The slight increase in the proportion of bed nets ownership in this study population might be attributed to the school bed net campaign piloted in this region to improve bed net coverage (33, 34). A high level of knowledge on malaria infection and interventions was significantly associated with the ownership of bed nets in this study. Studies from Ethiopia, Ghana, and Nigeria have also shown that high level of knowledge is associated with higher ownership of bed nets (8–12). However, similar to the findings in other studies (13–16, 35), in this study, the level of knowledge on malaria infection and intervention was not associated with bed net use.

The antenatal clinic (ANC) was the major source of the bed nets for most of the households in Masasi and Nanyumbu districts, followed by the mass bed nets distribution campaigns. Routine ANC services constitute an important delivery route of bed nets and it plays an important role in maintaining population-level coverage between campaigns, particularly for women who become pregnant between these health operations and for infants born outside of these periods (36). Furthermore, LLINs accounted for 67.00% of the bed nets, 7.90% were untreated bed nets, and the rest were unknown. The use of treated bed nets would be ideal, however, having untreated bed nets is better than nothing as it provides a physical barrier against the infected female *Anopheles* mosquitos. The major reasons hindering the ownership and use of bed nets in this study population included high bed nets cost (63.00%), and scarcity of bed nets (60.10%), as reported also in other parts of Tanzania (17). Normally, the free mass bed net distribution campaigns are conducted every 5 years. Reducing the interval between distribution campaigns may ensure that the population is well-covered with bed nets and overcome the issue of bed net costs and scarcity. The provision of more information to counteract misinformation may improve the bed net use in the population.

On the other hand, the majority (96.60%) of the household heads reported having slept under the bed net the previous night. In other parts of the country, a much lower proportion of participants reported having slept under the bed net the previous night (17). However, the level of knowledge on malaria infection was not associated with sleeping under the bed net. Conversely, only a few household heads did not sleep under the bed net the previous night, and lack of bed net was the major reason associated with the failure, followed by negligence and the perceived increased room temperature caused by the bed net use. Similar factors have been mentioned in other studies to be hindering the use of bed nets (15, 17). On the other hand, the majority of the household heads were comfortable sleeping under the bed net and also perceived that sleeping under the bed net had resulted in benefit of protection against mosquitoes. Similarly, almost all of them were ready to acquire bed nets from any sources just in case the ones that they had would become unusable. The good attitude toward malaria interventions might have been attributed to the adequate information on malaria infection and interventions the household heads have received through various sources, particularly the ANCs. In Tanzania, there is a campaign that encourages the husbands to escort their pregnant spouses to the clinic where health education including the importance of using bed nets is provided. Likewise, the school and free mass bed nets distribution campaigns are normally accompanied by the provision of health education on malaria infection and interventions including the benefit of using the bed nets. This may have contributed to the good attitude of the population toward malaria interventions.

## Study limitations

This study was based on quantitate design only and so could not explore in detail the perceptions of the community members in terms of knowledge, attitude, and practice related to the available malaria interventions in the Nanyumbu and Masasi districts. A social desirability bias can't be excluded, considering the mismatch between respondents confirming that they slept under a net (96.60%) and the lower presence of nets being hung on the sleeping spaces in the households (84.00%).

## Conclusion

Over all the study population showed a good level of knowledge about malaria and its interventions; the majority of them were using bed nets and presented a good attitude toward malaria interventions. The association between the level of knowledge and malaria infection in children under-five emphasizes the utility and need to continue with educational interventions as a preventive strategy.

## Data availability statement

The original contributions presented in the study are included in the article/Supplementary material, further inquiries can be directed to the corresponding author.

## Ethics statement

The studies involving human participants were reviewed and approved by the Muhimbili University of Health and Allied Sciences Ethics Committee. Written informed consent to participate in this study was provided by the participants' legal guardian/next of kin.

## Author contributions

AM, BM, BN, FC, FM, RM, and SL provided the conception and design of the study. BM, RM, BN, and BS collected data in the field. RM and BM performed data analysis and interpretation. RM drafted the manuscript together with BM and BN. All authors revised the manuscript critically for intellectual content and approved the final version.
